# Racial/Ethnic Disparities in Getting COVID-19 Vaccine: Do Age, Gender, and Education Matter?

**DOI:** 10.1089/heq.2022.0025

**Published:** 2022-07-04

**Authors:** Wei Zhang, Yan Yan Wu, Bei Wu

**Affiliations:** ^1^ Department of Sociology, University of Hawai'i at Mānoa, Honolulu, Hawaii, USA.; ^2^ Thompson School of Social Work & Public Health, University of Hawaiʻi at Mānoa, Honolulu, Hawaii USA.; ^3^ Rory Meyers College of Nursing, New York University, New York, New York, USA.; ^4^ NYU Aging Incubator, New York University, New York, New York, USA.

**Keywords:** COVID-19, vaccination rate, disparities, race/ethnicity, age, education

## Abstract

**Objectives:**

COVID-19 disproportionately affects racial/ethnic minorities and vaccine can help mitigate infection and transition, decrease rate of hospitalization, lower mortality rate, and control the pandemic. This study aims to examine disparities in COVID-19 vaccination rate by age among Whites, Hispanics, Blacks, and Asian Americans, and the modification effects by gender and education.

**Methods:**

We used seven waves of biweekly surveys from the Household Pulse Survey collected between July 21, 2021, and October 11, 2021.

**Results:**

Asians reported the highest, Blacks reported the lowest vaccination rate, and gender differences were minimal. Increasing age was associated with higher vaccination rate except for the oldest age group. The decline was from 84.4% (70–79 years) to 41.1% (80–88 years: 41.1%) among Hispanics and 92.8% to 69.6% among Asians. Educational effect was the most salient among younger adults with the largest gaps observed in Blacks. Among 18–29-year Black participants, the vaccination rates were 31.1% (confidence interval [95% CI]: 25.7–37.1) for high school or lower, 58.9% (95% CI: 54.2–63.5) for some college or associate degree, and 74.2% (95% CI: 69.4–78.5) for bachelor or higher degrees, leaving a 43.1% gap between the lowest and the highest education levels. The gaps in this age group were 33.7% among Whites, 32.1% among Hispanics, and 20.5% among Asian Americans.

**Conclusion:**

Our study advances the existing literature on COVID-19 vaccination by providing empirical evidence on the dynamic race/ethnic–age–education differences across racial/ethnic groups. The findings from our study provide scientific foundation for the development of more strategies to improve vaccination rate for the minority populations.

## Introduction

COVID-19 disproportionately affects racial/ethnic minorities with regard to number of infected cases, hospitalizations, and death^1–3^ compared with non-Hispanic Whites.^4–8^ Vaccination can help mitigate SARS-CoV-2 infection and transition, decrease rate of hospitalization, lower mortality rate,^9^ establish community/herd immunity (75–85% of the population vaccinated),^10^ and control the pandemic. In a recent survey, ∼67% of the U.S. population reported willingness to receive the COVID-19 vaccine^11^ and this percentage has been increasing over time.^12,13^

However, studies also showed that vaccine hesitancy was prevalent among racial/ethnic minorities such as Black communities in the United States^14,15^ whereas Asians generally reported the highest acceptance rate of COVID-19 vaccine.^16,17^ The vaccination acceptance, in turn, is closely related to the actual vaccination status. For instance, the latest data^18^ showed that the vaccination rate among the Asian, White, Hispanic, and Black populations was 85%, 63%, 65%, and 57%, respectively.

Vaccination hesitancy within some racial/ethnic minorities was largely due to societal and cultural barriers^10^ and lack of trust.^14^ For instance, using focus group data, one study^15^ identified the possible drivers to vaccine hesitancy among Blacks included mistrust in the medical establishment, concerns about the accelerated timeline for vaccine development, limited data on the possible side effects, and political environment promoting racial injustice. Disparities in vaccine hesitancy may influence vaccination rate and accelerate the existing racial/ethnic disparities in COVID-19 infection, hospitalization, and mortality rates. It is important to examine racial/ethnic disparities in the actual vaccination rate using large national survey data.

Besides racial/ethnic disparities, studies also found that demographics and socioeconomic status such as higher levels of education and income, as well as old age were associated with higher levels of vaccine acceptance^17,19^ as well as the actual vaccination rate.^20^ These findings are not very surprising as socioeconomic privilege such as education and income often lead to healthier behaviors through pathways of health literacy, social support, and locus of control in particular.^21,22^ And compared with older adults, younger adults (i.e., 18–49 years) were found to progressively become less likely to state that they would get a COVID-19 vaccine.^23^ These age disparities might be largely due to mistrust and lack of underlying health conditions among younger age groups.

Besides education and age, findings on gender disparities in vaccination hesitancy and acceptance are somewhat puzzling. Literature suggests that women are more likely to use health care services than men.^24–26^ When it comes to the COVID-19 pandemic, women were also found to be more likely to perceive it as a very serious health problem and to comply with restrictive public policy measures than men.^27^ However, studies have started to show that women often reported more concerns about impact on fertility or pregnancy, thus lower willingness to take COVID-19 vaccine than their male counterparts.^14,17,19^ So, it is worth examining whether this gender disparity in COVID-19 hesitancy will reflect in the actual vaccination uptake. Collectively, age, education, and gender are likely to moderate the association between race/ethnicity and the vaccination rate.

In summary, despite numerous studies available to examine disparities in vaccination hesitancy, acceptance, and willingness, very few studies have used nationally representative data to systematically examine the actual vaccination rate among various social groups. Our study aims to examine racial/ethnic disparities in getting COVID-19 vaccine and to explore the effects of age, gender, and education in moderating the association between race/ethnicity and the prevalence of vaccination using multiple phases of the Household Pulse Survey (HPS).

Given Asians' highest level of vaccination acceptance revealed in previous studies, we first hypothesize that Asians have the highest rate of vaccination, in comparison with other racial/ethnic groups including Whites, Hispanics, and Blacks. We then hypothesize that age, gender, and education may modify the association between race/ethnicity and rate of vaccination.

## Materials and Methods

### Data

Launched in April 2020, the HPS^28^ is an ongoing cross-sectional survey developed by the U.S. Census Bureau and the National Center for Health Statistics to measure household experiences during the coronavirus pandemic. The Census Bureau's master address file was used to select a sample of U.S. households, and one adult per household (18–88 years) was recruited to answer a 20-min survey that included questions about education, employment, food sufficiency, housing security, physical and mental health, vaccination status, etc.

This study utilized seven waves of biweekly surveys from the Phase 3.2 data collection, from July 21, 2021, to October 11, 2021. The response rate ranged from 5.4% to 6.1% across the seven waves of the data collection. The total sample size is 369, with an analytic sample of 321 for the seven waves after removing multiracial respondents and respondents with other races. There are no missing values. Data are publicly available and de-identified, therefore the study is IRB exempted.

### Measures

The response variable, vaccination status, was determined by two questions: “have you received at least one dose of a COVID-19 vaccine” and “did you receive (or do you plan to receive) all required doses?” Those who answered yes to both were coded as yes and the rest were coded as no. Our explanatory variables are age; gender (male, female); race/ethnicity (single race White, Black, Hispanic, and Asian); and education (high school, high school equivalent or lower, some college or associate degree, and bachelor degree or higher). Age is categorized into 10-year groups except for the youngest (18–29 years) and the oldest groups (80–88 years).

### Statistical analysis

Statistical software R version 4.0.5 and library “survey” were used for the analysis. All analysis accounted for survey weights with each survey wave weighted equally. We computed weighed descriptive statistics to summarize sample characteristics and the vaccination rates by race/ethnicity. Next, we performed a series of weighted logistic regression analysis and examined the interaction effects between all explanatory variables. Final models are based on the judgment of both statistical and practical significance. To illustrate the differences by explanatory variables, we calculated the weighted rates of vaccination and the confidence intervals (95% Cis) based on the final models and presented the results in figures.

## Results

### Sample characteristics

Table 1 gives sample characteristics by race/ethnicity. The weighted percentage of males are 48.7% in Whites, 50% in Hispanics, 43.6% in Blacks, and 52.3% in Asian Americans. The proportion of bachelor degree or higher is 33.7% in Whites, 18.3% in Hispanics, 24% in Blacks, and 55.5% in Asian Americans.

**Table 1.** Sample Characteristics (*N*: Sample Size, wt%: Weighted Percentage) by Race/Ethnicity (*N*=369,321)

	White		Hispanics		Black		Asian	
	*N*	wt%	*N*	wt%	*N*	wt%	*N*	wt%
Total by race/ethnicity	285,213	77.2	35,990	9.7	29,365	8.0	18,753	5.1
Gender								
Male	116,252	48.7	14,252	50.0	9201	43.6	9530	52.3
Female	168,961	51.3	21,738	50.0	20,164	56.4	9223	47.7
Age group (years)								
18–29	21,146	16.8	5258	25.6	2383	14.7	2408	23.0
30–39	42,356	16.7	7535	21.4	5064	20.2	3868	21.7
40–49	48,561	14.8	8075	19.7	6508	20.8	4425	19.6
50–59	55,397	17.0	7011	15.7	6649	20.8	3749	17.5
60–69	67,187	20.0	5210	11.0	5909	16.0	2693	11.8
70–79	41,460	11.7	2165	4.2	2500	6.4	1350	5.0
80–88	9106	2.9	736	2.4	352	1.1	260	1.3
Education								
High school or lower	34,324	35.5	8368	53.0	5140	43.8	1272	22.4
Some college or associate	89,434	30.7	13,157	28.6	11,085	32.2	3516	22.1
Bachelor or higher	161,455	33.7	14,465	18.3	13,140	24.0	13,965	55.5

### Vaccination rates by demographics and race/ethnicity

The weighted vaccination rates and 95% Cis by race/ethnicity and demographics are listed in Table 2. The overall rates are 77.8% (95% CI: 77.5–78.2) in Whites, 73.5% (95% CI: 72.5–74.5) in Hispanics, 70.8% (95% CI: 69.7–71.9) in Blacks, and 90.5% (89.5–91.5) in Asian Americans. Gender difference were minimal, ranging from 0.5% to 1.5% among the four racial/ethnic groups. The vaccination rates were the highest among those who had bachelor or higher degree: 89.1% (95% CI: 88.8–89.3) among Whites, 83.4% (95% CI: 82.3–84.4) among Hispanics, 84.9% (95% CI: 83.9–85.9) among Blacks, and 94.9% (95% CI: 94.3–95.5) among Asian Americans.

**Table 2.** Weighted Vaccination Rates (wt%) and 95% Confidence Intervals by Demographics and Race/Ethnicity

Demographics	White	Hispanics	Black	Asian
	wt% (95% CI)	wt% (95% CI)	wt% (95% CI)	wt% (95% CI)
Overall	77.8 (77.5–78.2)	73.5 (72.5–74.5)	70.8 (69.7–71.9)	90.5 (89.5–91.5)
Gender				
Male	77.1 (76.5–77.6)	72.9 (71.3–74.5)	71.8 (69.8–73.7)	90.3 (88.7–91.6)
Female	78.5 (78.1–78.9)	74.1 (72.9–75.3)	70.0 (68.8–71.2)	90.8 (89.4–92.1)
Age group (years)				
18–29	68.6 (67.4–69.8)	67.6 (65.1–70.0)	48.7 (45.2–52.2)	89.3 (86.2–91.7)
30–39	70.9 (70.0–71.8)	68.7 (66.4–70.9)	54.7 (52.3–57.2)	89.6 (87.4–91.4)
40–49	72.4 (71.5–73.2)	75.9 (74.0–77.8)	70.7 (68.4–72.9)	91.6 (89.5–93.3)
50–59	79.6 (78.9–80.3)	80.4 (78.2–82.4)	81.0 (78.7–83.1)	92.9 (91.2–94.3)
60–69	86.0 (85.4–86.6)	85.3 (83.3–87.1)	89.0 (87.4–90.4)	91.1 (88.5–93.1)
70–79	89.5 (88.8–90.2)	84.4 (80.4–87.8)	90.2 (87.7–92.3)	92.8 (88.7–95.4)
80–88	84.4 (82.2–86.4)	41.1 (33.6–49.1)	89.1 (81.3–93.9)	69.6 (49.9–84.0)
Education				
High school or lower	68.4 (67.6–69.3)	68.3 (66.6–70.0)	62.3 (60.1–64.4)	80.3 (76.4–83.7)
Some college or associate	76.3 (75.8–76.8)	76.9 (75.6–78.1)	71.9 (70.5–73.2)	89.9 (88.0–91.6)
Bachelor or higher	89.1 (88.8–89.3)	83.4 (82.3–84.4)	84.9 (83.9–85.9)	94.9 (94.3–95.5)

The chi-square tests of association between vaccination and age, and vaccination and education are statistically significant with *p*-values <0.001. Gender is only statistically associated with vaccination Whites (*p*<0.001) due to large sample size with a 1.4% gender difference.

CI, confidence interval.

In general, increasing age was associated with higher vaccination rates except for the oldest age group. The decline was from 84.4% in 70–79-year-age group to 41.1% in 80–88-year-age group among Hispanics, and from 92.8% (70–79 years) to 69.6% (80–88 years) among Asian Americans. The decline was from 89.5% to 84.4% among Whites, and from 90.2% to 89.1% among Blacks.

### Education–age–race/ethnicity interaction

**FIG. 1.**
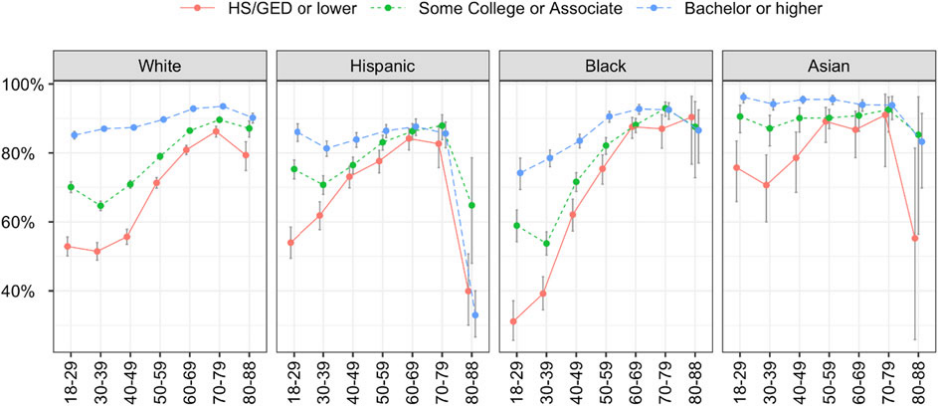
Weighted vaccination rates and 95% CI by age, education, and race/ethnicity. CI, confidence interval.

There is a strong three-way statistical interaction effects between age, education, and race/ethnicity (*p*<0.0001). The weighed prevalence and 95% CI by the three variables are listed in Table 3. As illustrated in Figure 1, among the 18–29-year-old Black respondents, the vaccination rates were 31.1% (95% CI: 25.7–37.1) for high school or lower, 58.9% (95% CI: 54.2–63.5) for some college or associate degree, and 74.2% (95% CI: 69.4–78.5) for the bachelor or higher degrees, leaving a 43.1% gap between the lowest and the highest educational levels. The gaps in this age group were 33.7% in Whites, 32.1% in Hispanics, and 20.5% in Asian Americans.

**Table 3.** Weighted Vaccination Rates (wt%) and 95% Confidence Interval by Age, Education, and Race/Ethnicity

Age group (years)	White	Hispanic	Black	Asian
	wt% (95% CI)	wt% (95% CI)	wt% (95% CI)	wt% (95% CI)
18–29				
High school or lower	52.9 (50.1–55.6)	54.0 (49.4–58.5)	31.1 (25.7–37.1)	75.7 (65.9–83.5)
Some college or associate	70.1 (68.5–71.6)	75.3 (72.5–77.9)	58.9 (54.2–63.5)	90.6 (85.9–93.8)
Bachelor or higher	85.2 (84.0–86.2)	86.1 (83.3–88.5)	74.2 (69.4–78.5)	96.2 (94.5–97.4)
30–39				
High school or lower	51.4 (48.9–54.0)	61.9 (57.7–65.8)	39.2 (34.5–44.1)	70.7 (60.0–79.4)
Some college or associate	64.7 (63.3–66.0)	70.7 (68.0–73.4)	53.7 (50.4–57.1)	87.1 (82.0–90.9)
Bachelor or higher	87.0 (86.4–87.6)	81.3 (79.0–83.4)	78.5 (76.0–80.9)	94.2 (92.4–95.5)
40–49				
High school or lower	55.7 (53.5–57.9)	73.1 (69.9–76.1)	62.1 (57.4–66.6)	78.6 (68.5–86.0)
Some college or associate	70.9 (69.7–72.0)	76.5 (74.0–78.7)	71.6 (68.8–74.2)	90.1 (86.0–93.1)
Bachelor or higher	87.4 (86.8–88.0)	83.9 (81.7–85.9)	83.5 (81.5–85.4)	95.5 (94.4–96.4)
50–59				
High school or lower	71.3 (69.8–72.9)	77.6 (74.2–80.7)	75.4 (70.9–79.4)	89.1 (83.1–93.2)
Some college or associate	79.0 (78.1–79.9)	83.1 (80.8–85.1)	82.1 (79.6–84.4)	90.2 (87.1–92.6)
Bachelor or higher	89.7 (89.2–90.2)	86.4 (84.4–88.2)	90.6 (88.9–92.0)	95.5 (94.1–96.7)
60–69				
High school or lower	80.9 (79.5–82.2)	84.2 (80.9–87.0)	87.6 (84.2–90.3)	86.7 (78.6–92.1)
Some college or associate	86.5 (85.8–87.1)	86.3 (83.8–88.4)	88.1 (85.9–90.0)	90.8 (86.3–94.0)
Bachelor or higher	92.8 (92.4–93.2)	87.6 (85.1–89.8)	92.8 (91.2–94.0)	94.0 (92.1–95.4)
70–79				
High school or lower	86.2 (84.6–87.7)	82.7 (75.7–87.9)	87.0 (81.4–91.1)	91.0 (76.0–97.0)
Some college or associate	89.6 (88.8–90.4)	87.9 (83.8–91.1)	93.0 (90.6–94.8)	92.6 (86.1–96.2)
Bachelor or higher	93.5 (93.0–94.0)	85.6 (81.5–89.0)	92.5 (89.8–94.6)	93.8 (89.7–96.4)
80–88				
High school or lower	79.4 (74.9–83.2)	39.9 (30.0–50.7)	90.4 (76.7–96.4)	55.2 (25.9–81.4)
Some college or associate	87.1 (84.6–89.3)	64.8 (48.0–78.6)	87.6 (72.8–94.9)	85.3 (56.4–96.3)
Bachelor or higher	90.2 (88.7–91.5)	32.9 (26.6–40.0)	86.6 (77.1–92.5)	83.3 (69.8–91.4)

## Discussion

Using the nationally representative data in the United States, this study revealed significant racial/ethnic, educational, and age disparities in COVID-19 vaccination rate. This pattern is consistent with prior research on COVID-19 vaccine acceptance using a nationwide U.S.-based survey.^17^ Overall, Asians reported the highest vaccination rate followed by Whites, Hispanics, and Blacks. This finding is supported by a previous study^29^ on COVID-19 vaccine intentions, revealing that Asian/Pacific islanders had the highest probability of likely getting a COVID-19 vaccine. Cultural norms and attitudes may play a major role in explaining these differences.

In general, Asian cultures largely emphasize compliance to health authority and collectivisms over individualism. Better access to care is another reason. Based on the census and CDC data, Asians, on average, have higher levels of socioeconomic status than other racial/ethnic groups.^30,31^ Most Asians also heavily reside in urban areas. Therefore, they have better access to vaccination with fewer geographical barriers. In addition, Asians were found to have the higher trust in the scientific community compared with other racial/ethnic groups.^32^ Another important factor is that Asian Americans, in general, have higher health status than other racial/ethnic groups. Studies^33^ showed that those who reported higher health status were less likely to have vaccine concerns than those with lower health status.

In contrast, however, we should keep in mind that Asian American is a diverse population and the seemly highest vaccination rate might, to some extent, conceal the within group differences. For instance, one recent study^33^ revealed that, before the first vaccine became available in December 2021, some Asian groups such as Vietnamese American showed less concerns compared with Chinese, Filipino, and Korean Americans. Their findings suggest the potential within-group heterogeneity among Asian Americans regarding vaccination acceptance warrants further exploration.

Our study also revealed a counterintuitive finding: Although the oldest old had the highest rates of COVID-19 mortality and hospitalization,^3^ these community-dwelling older adults had a lower rate of vaccination than other young older adults (65+ years). This finding is particularly shown in Asians and Hispanics who had low levels of education. This finding is concerning as older age is one of the leading risk factors for COVID-19 mortality and complications. Previous research reported lower vaccine willingness in women than in men,^19^ our studies found no gender differences in actual vaccine uptake using most recent survey data.

This finding may indicate that people's attitude, women in particular, is dynamic and it may be affected by increasing availability of health information. For instance, there are at least two studies^12,13^ surveyed changes in acceptance of the COVID-19 vaccine and both studies found that the acceptance rate has been increasing from 2020 to 2021.

In addition, education was found to be an important correlate of vaccination uptake: Except for the oldest old (80+ years), education is positively related to vaccination uptake for all racial/ethnic groups and all age groups. This finding is consistent with the literature on COVID-19 vaccine hesitancy/acceptance.^19^ It is not surprising that the level of education is highly associated with health literacy defined as “the capacity to obtain, process, and understand basic health information and services needed to make appropriate health decisions to adhere to disease-management protocols.”^21^ Health literacy, in turn, is related to health behaviors such as smoking, drinking, and exercising,^34^ and plays an important role in making a vaccination decision.^35^

However, the education pattern on vaccination uptake was found to be in the opposite when the oldest old were examined in Blacks, Asians, and Hispanics groups. For Hispanics, the vaccination rate among those with highest level of education is the lowest for respondents aged 80 years and above. This finding is quite puzzling and it may be due to small sample sizes for the oldest-old age group as well as the survival selection effect. More research, perhaps qualitative focus group studies are need to examine barriers to vaccination update among the oldest-old minorities.

There are a few limitations in the survey data that are worth noting. First, the survey data were collected in English, thus, individuals (within the Asian and Hispanic populations) with limited English proficiency were systematically excluded from the study. Second, the short duration of the data collection may miss the changing dynamic in vaccination acceptance over time. Third, we were unable to capture the within-group heterogeneity due to lack of ethnic data within each specific group. In the future, more studies are needed to examine the trend of vaccination rate over time along with the recent development of COVID-19 variants (e.g., delta and omicron) as well as to examine differences in rate within each of the racial/ethnic groups.

Despite these limitations, our studies have important policy implications: More efforts are needed to improve health literacy for the general population and more emphasis needs to be given to racial/ethnic minority populations such as Blacks, Hispanics, as well as and the oldest-old age group. For instance, given that the oldest old may have limited abilities in searching for and comprehending the complex and evolving COVID-19–related health information, family members or caregivers should be contacted and educated so that they can convey useful messages to the oldest old, helping them make informed decision regarding vaccine uptake.

It is also critical to work with community organizations to promote health education and build trust toward vaccination for minority populations. In addition, given that the effect of education on vaccination rate varies by race/ethnicity and age groups, access barriers need to be identified and tailored vaccination promotion programs need to be developed for different target populations.

## Conclusion

In conclusion, consistent with prior research on vaccination hesitancy/acceptance, our study has provided empirical evidence on racial/ethnic disparities in vaccination uptake, revealing that Asians reported the highest, Blacks reported the lowest vaccination rate, and Whites and Hispanics are in between. Our study advances the existing literature on COVID-19 vaccination by demonstrating the dynamic race/ethnic–age–education differences. Age differences in vaccination rate were minimal among Asians but substantial among Blacks. The positive effects of education are more salient for Whites and Asians than for Blacks and Hispanics. Especially for Hispanics aged 70 years and above, the effects of education are minimal.

The findings from our study provide scientific foundation for the development of more strategies to improve vaccination rate for minority populations. More programs and services are needed to promote vaccination rate among young and older population, with minority populations in particular. These interventions can include involvement of family caregivers, school-based vaccination programs, and working with community organizations (e.g., senior centers, adult day-care centers, meals on wheels programs) to improve education and vaccination rate to community-dwelling older adults. For home-bound older adults, vaccination can be delivered at their home by working with visiting nurses' associations.

## Abbreviation Used

CIs: confidence intervals
